# Gut microbiota differs in composition between adults with type 1 diabetes with or without depression and healthy control participants: a case-control study

**DOI:** 10.1186/s12866-022-02575-1

**Published:** 2022-06-28

**Authors:** Frank Petrak, Stephan Herpertz, Julia Hirsch, Bonnie Röhrig, Iris Donati-Hirsch, Georg Juckel, Juris J. Meier, Sören Gatermann

**Affiliations:** 1 Department of Psychosomatic Medicine and Psychotherapy, LWL-University Clinic Bochum, Ruhr-University Bochum, Bochum, Germany; 2 Center for Psychotherapy Wiesbaden, Wiesbaden, Germany; 3 Diabetology practice, Dortmund, Germany; 4 Department of Psychiatry, LWL-University Hospital, Ruhr-University Bochum, Bochum, Germany; 5 Department of Internal Medicine, Gastroenterology and Diabetology, Augusta Clinic Bochum, Bochum, Germany; 6 German National Reference Center for Multidrug-Resistant Gram-Negative Bacteria, Department of Medical Microbiology, Ruhr-University Bochum, Bochum, Germany

**Keywords:** Abundances, Alpha diversity, Beta diversity, Depression, Diabetes mellitus type 1, Gut microbiota, Veillonellaceae, Megasphaera

## Abstract

**Background:**

Individuals with type 1 diabetes and those with depression show differences in the composition of the gut microbiome from that of healthy people. However, these differences have not yet been studied in patients with both diseases. Therefore, we compared the gut microbiome of people with type 1 diabetes with or without depression with matched healthy controls.

**Methods:**

A case-control study was conducted in 20 adults with type 1 diabetes (group A), 20 adults with type 1 diabetes and depression (group B), and 20 healthy adults (group C). Gut microbiota composition was determined by sequencing of the V3-V4 region of the bacterial 16S rDNA and alpha and beta diversity was compared between the groups.

**Results:**

Groups A and B both showed higher alpha diversity than the healthy control group (*P* < 0.001) but alpha diversity did not differ significantly between groups A and B. Participants having type 1 diabetes with (*P* < 0.05) or without comorbid depression (*P* < 0.001) differed regarding beta diversity from healthy controls but not between each other. Group B (diabetes with depression) had significantly higher abundance of *Megaspaera* than groups A and C. Both diabetes groups had a higher abundance of *Christensenellaceae, Succinivibrionaceae*, and *Rhodospirillaceae* than the healthy group but similar between-group abundances.

**Conclusions:**

While differences in alpha and beta diversity and in some bacterial taxa occurred only between participants with diabetes and healthy controls, specific characteristics regarding the abundance of *Megasphaera* were observed in people with diabetes and comorbid depression. In summary, the study findings indicate a possible involvement of bacterial groups in depression in people with diabetes. The results suggest replication studies in larger samples to verify these findings.

## Background

The microbiome describes the totality of microorganisms on the surfaces of the human body and has increasingly become a subject of scientific interest as a factor of mental and somatic health. There is increasing evidence that intestinal dysbiosis is associated with various diseases, including type 1 and type 2 diabetes [1]. While the evidence for the functional association of the gut microbiome with type 2 diabetes continues to grow almost exponentially [2, 3], the data for type 1 diabetes are limited. Nevertheless, there is evidence that patients with type 1 diabetes have a less diverse and less stable gut microbiome than healthy individuals, such as observations of changes in the ratio of Firmicutes to Bacteroidetes [4, 5]. Case-control study results showed that people with type 1 diabetes had significantly lower microbiota diversity than healthy participants, with a significantly higher relative abundance of the genera *Bacteroides, Ruminococcus, Veillonella, Blautia*, and *Streptococcus* and a lower relative abundance of *Bifidobacterium, Roseburia, Faecalibacterium*, and *Lachnospira* [6].

In addition to the described association with somatic diseases, the influence of the gut microbiome on various mental disorders, especially depressive disorders, is being increasingly investigated [4, 5]. For example, the “leaky gut hypothesis” already invoked in type 1 diabetes has also been postulated in depression. It is assumed that a disturbed intestinal barrier function results in an impairment of the immune system and promotes various inflammatory processes, which in turn play an important role in the development of depression [6]. The results from a large microbiome cohort described the characteristic peculiarities of the gut microbiome of depressed participants, with lower abundances of *Coprococcus* and *Dialister* [4]. While the common biological mechanisms of type 2 diabetes and depression are becoming increasingly well understood, the corresponding evidence for type 1 diabetes remains limited [7, 8]. In summary, findings on the role of the gut microbiome in both depressive disorders and diabetes suggest that the corresponding interactions may also play a role in the comorbidity of diabetes and depression. To date, evidence on the gut microbiome in the pathogenesis of both diabetes and depression is largely unrelated, and to our knowledge, the characteristics of the gut microbiome of patients with diabetes and comorbid depression have been unexplored.

Against this background, this case-control study investigated how the gut microbiome of participants with type 1 diabetes and comorbid depression differs from the gut microbiome of nondepressed participants with type 1 diabetes and that of healthy control group. Differences in alpha and beta diversity and in the abundance of individual bacterial taxa were analyzed.

## Methods

The study was designed as a noninterventional cross-sectional case-control study. Three different groups of participants were studied regarding the characteristics of their gut microbiome:

- 20 participants with type 1 diabetes and comorbid moderate to severe depressive episodes
- 20 participants with type 1 diabetes without depression
- 20 healthy individuals (without diabetes and without depression)

After a screening and diagnostic phase, a psychometric questionnaire examination was administered, and stool and blood samples were collected. In addition, an electroencephalography (EEG) examination to derive loudness-dependent auditory evoked potentials (LDAEP) was performed as part of a secondary question of the study reported elsewhere (Flasbeck V, Hirsch J, Petrak F, Meier J, Herpertz S, Gatermann S, et al: Microbiome composition and central serotonergic activity in patients with depression and type 1 diabetes, submitted).

### Study participants, inclusion, and exclusion criteria

Participants were recruited in a diabetological practice in Dortmund and an outpatient diabetes department of a clinic in Bochum (both in Germany) from December 2018 through October 2019. Patient medical records were consulted to select potentially eligible participants with type 1 diabetes. To ensure the diagnosis of type 1 diabetes, only patients whose diagnoses dated back at least six months and who were treated with ≥20 insulin units per day were selected. All patients were informed and enrolled in the study if all other requirements were met (further eligibility criterion: 18 to 65 years of age).

An additional inclusion criterion for participants with depression was a diagnosis of moderate or severe depression according to the criteria of the Fifth Edition of the Diagnostic and Statistical Manual of Mental Disorders (DSM-5) [9]. After depression screening with a cutoff sum score ≥ 5 from the Patient Health Questionnaire (PHQ-9) [10], a diagnosis of depression for screening-positive participants was verified with the Diagnostic Interview for Mental Disorders (DIMD-short version) [11], a semistructured interview that allows the diagnosis of mental disorders according to the DSM-5.

The inclusion criterion for the healthy control group was only an age between 18 and 65 years. To minimize the effect of possible confounding variables, the control group was matched for the variables sex, age, and hormonal contraception.

A comprehensive set of exclusion criteria was established and reviewed by the treating physician. Exclusion criteria for all study participants: antibiotic treatment 6 months prior to stool sample collection; the presence of any of the following 3 months prior to stool sample collection: gastroenteritis, colon hydrotherapy, *Clostridium difficile* infection, or pregnancy; and insufficient knowledge of the German language. Additional exclusion criteria for participants with type 1 diabetes with or without comorbid depression: celiac disease, chronic diarrhea, chronic constipation, gastroparesis, nephropathy, proliferative retinopathy, polyneuropathy, macroalbuminuria, inflammatory bowel disease, severe other physical illness, mental comorbidities, regular laxative use, antidepressant medication, and any other medical condition or medical treatment that, in the judgment of a member of the study group (physician or psychologist), was incompatible with study participation. Exclusion criteria for the healthy group: known severe physical illness, PHQ score ≥ 5 in the depression screening and further exclusion of a previously unrecognized mental disorder using the DIMD-short version. The recruitment of healthy volunteers for the control group was done through various means of public relations (personal contacts of the study staff, online information, and notices posted at Bochum University). All people who participated in the study received a compensation of €100.

### Further questionnaires

To control for dietary factors, participants were asked about their dietary habits (‘no special diet, vegetarian diet, vegan diet, or other diet’).

Additional psychometric questionnaires on generic and diabetes-specific distress were administered to analyze further research questions in the context of another planned publication. This included the Trier Inventory of Chronic Stress (TICS-SSCS) [12] and the Problem Areas in Diabetes Questionnaire (PAID) [13] for participants with type 1 diabetes, with a cutoff sum score ≥ 40 indicating severe diabetes distress. The results of the TICS-SSCS and the PAID are presented in this publication only regarding descriptive statistics and tests of mean differences between the three groups examined (see Table 1).

**Table 1:** Characteristics of the participants and differences in the composition of gut microbiota between groups

Characteristics	Type 1 diabetes (***n*** = 20)	Type 1 diabetes and depression (***n*** = 20)	Healthy control group (***n*** = 20)	Total sample (***N*** = 60)
Age (in years)	42.20 ± 15.05	43.45 ± 12.07	41.85 ± 14.15	42.19 ± 13.89
Female sex	13 (65.0%)	13 (65.0%)	13 (65.0%)	39 (65.0%)
Years of formal education**				
0–10	4 (20.0%)	13 (65.0%)	3 (15.0%)	20 (33.3%)
> 10	16 (80.0%)	7 (35.0%)	17 (85.0%)	40 (66.7%)
Diabetes duration (in years)	16.20 ± 12.56	20.75 ± 13.09	–	–
Body-Mass-Index (BMI)**	23.76 ± 2.65	27.15 ± 4.18	24.19 ± 2.72	25.03 ± 3.55
Smoking (yes)*	2 (10.0%)	6 (30.0%)	1 (5.0%)	9 (15.0%)
Dietary habits				
No special diet	18 (90.0%)	14 (70.0%)	17 (85.0%)	49 (81.7%)
Vegetarian	2 (10.0%)	2 (10.0%)	2 (10.0%)	6 (10.0%)
Vegan	0 (0.0%)	0 (0.0%)	0 (0.0%)	0 (0.0%)
Other diets	0 (0.0%)	4 (20.0%)	1 (5.0%)	5 (8.3%)
Contraceptives (yes)	4 (20.0%)	2 (10.0%)	4 (20.0%)	10 (16.7%)
Further somatic diseases (yes)**	13 (65.0%)	18 (90.0%)	9 (40.0%)	40 (66.7%)
Arterial hypertension	4 (20.0%)	4 (20.0%)	4 (20.0%)	12 (20.0%)
Thyroid diseases**	8 (40.0%)	11 (55.0%)	2 (10.0%)	21 (35.0%)
Hypercholesterolemia	4 (20.0%)	4 (20.0%)	0 (0.0%)	8 (13.3%)
High-sensitivity C-reactive protein (hs-CRP (mg/dl))	1.59 ± 2.70	2.13 ± 2.66	2.28 ± 3.88	2.00 ± 3.11
Major depression (Mini-DIPS)				
Single episode	–	5 (25.0%)	–	–
Recurrent episodes	–	15 (75.0%)	–	–
Severity of depression (PHQ-9, range 0–27)***	6.05 ± 3.75	14.15 ± 3.54	1.80 ± 1.24	7.33 ± 5.98
TICS-SSCS (range 0–48)***	13.25 ± 9.78	27.80 ± 7.04	8.15 ± 4.66	16.40 ± 11.15
PAID sum score (range 0–100)***	20.55 ± 17.28	41.81 ± 15.31	–	–
**Alpha diversity: Differences in the composition of gut microbiota between groups**				
Number of species***	747.55 ± 44.30	737.75 ± 55.96	647.00 ± 83.21	711.85 ± 76.59
Shannon index***	4.59 ± 0.20	4.61 ± 0.22	4.18 ± 0.45	4.47 ± 0.36
ACE‡	807.17 ± 46.82	800.60 ± 59.09	711.60 ± 86.24	774.16 ± 77.90
CHAO1*	788.59 ± 44.98	785.42 ± 61.22	702.20 ± 90.01	759.70 ± 77.49
**Abundances of different taxonomic levels (numbers and relative abundance): Differences between groups** (only significant results)				
** Phyla**				
Firmicutes				
Numbers Relative abundance	41,950 ± 7755 0.39 ± 0.07	44,022 ± 9014 0.41 ± 0.08	37,190 ± 7583 0.34 ± 0.07	41,120 ± 8507 0.38 ± 0.08
Bacteroidetes				
Numbers Relative abundance	56,430 ± 8806 0.52 ± 0.08	52,814 ± 8981 0.49 ± 0.08	59,305 ± 8434 0.55 ± 0.08	56,130 ± 9002 0.52 ± 0.08
Actinobacteria				
Numbers Relative abundance	383 ± 345 0.0035 ± 0.0032	446 ± 220 0.0041 ± 0.0020	544 ± 294 0.0063 ± 0.0027	456 ± 293 0.0042 ± 0.0027
** Orders**				
Lactobacillales				
Numbers Relative abundance	93 ± 58 9e-4 ± 5e-4	117 ± 139 1e-3 ± 1.2e-3	1023 ± 971 9e-3 ± 9e-3	401 ± 698 4e-3 ± 6e-3
** Families**				
Veillonellaceae (Phylum Firmicutes)*				
Numbers Relative abundance	934 ± 569 0.006 ± 0.004	2647 ± 2228 0.02 ± 0.01	1770 ± 2487 0.01 ± 0.02	1784 ± 2039 0.01 ± 0.01
Christensenellaceae (Phylum Firmicutes)***				
Numbers Relative abundance	2268 ± 940 0.01 ± 0.006	1935 ± 949 0.01 ± 0.006	834 ± 799 0.005 ± 0.005	1693 ± 1082 0.01 ± 0.007
Lactobacillaceae (Phylum Firmicutes)***				
Numbers Relative abundance	19 ± 11 0.0001 ± 0.00007	55 ± 177 0.0003 ± 0.001	741 ± 721 0.005 ± 0.004	264 ± 531 0.001 ± 0.003
Leuconostocaceae (Phylum Firmicutes)***				
Numbers Relative abundance	0.35 ± 1 0.000002 ± 0.000006	1,6 ± 3.7 0.00001 ± 0.00002	288 ± 493 0.002 ± 0.003	93 ± 306 0.0006 ± 0.002
Succinivibrionaceae (Phylum Proteobacteria)***				
Numbers Relative abundance	1668 ± 3551 0.01 ± 0.02	3326 ± 5454 0.02 ± 0.03	75 ± 326 0.0005 ± 0.003	1717 ± 3960 0.01 ± 0.02
Rhodospirillaceae (Phylum Proteobacteria)*				
Numbers Relative abundance	1995 ± 1585 0.01 ± 0.01	1998 ± 1494 0.01 ± 0.01	928 ± 1632 0.005 ± 0.007	1652 ± 1623 0.01 ± 0.01

Note: Plus-minus values are means ± standard deviation; higher PAID scores indicate more diabetes related stress; higher PHQ-9 scores indicate higher depression severity; higher TICS-SSCS scores indicate higher general stress level; higher Shannon index, ACE and CHAO1 indicate higher alpha diversity

* = significant differences between groups *P* < 0.05

** = significant differences between groups *P* < 0.01

*** = significant differences between groups *P* < 0.001

### Microbiota analysis

Fecal samples were collected in stool transport tubes (StorAX, Axon Lab AG, Switzerland) and returned within 48 hours to the study center. After centrifugation at 8000×*g* for 60 s, the supernatant was discarded. Approximately 200 mg (wet weight) of pellets was frozen and stored at − 80 °C.

### DNA isolation and detection

A commercially available kit (QIAamp Fast DNA Stool Mini Kit, Qiagen, Germany) was employed to isolate the DNA of the intestinal microbiome in accordance with the manufacturer’s specifications. To homogenize the stool sample, approximately 200 mg of frozen stool was diluted in 1 ml of Inhibitex buffer and vortexed until the sample was fully homogenized. To remove the inhibitors and pelleted stool particles, the samples were incubated at 95 °C for 5 min and centrifuged for 1 min. Afterwards, 400 μLμL of supernatant was mixed with 30 μLμL of proteinase K and 400 μL of buffer AL and incubated for 10 min at 70 °C. Next, 400 μL of ethanol was added, and the samples were loaded into the QIAamp Spin Colum and centrifuged at 15,000 g for 1 min. The DNA was then washed twice and eluted with 100 μL of elution buffer. DNA concentrations were measured using a Qubit 1x HS dsDNA Assay Kit (Invitrogen, ThermoFisher Scientific, Germany), and checks for damage and degradation were additionally performed using agarose gel (0.8%) electrophoresis. Samples had a mean DNA concentration of 27.1 ng/μL. Then, samples were checked for bacterial 16S ribosomal RNA (rDNA) by polymerase chain reaction (PCR, AmpliTaq Gold Master mix, Applied Biosystem, ThermoFisher Scientific, Germany) using the primers 16s_27F 3′-AGAGTTTGATCMTGGCTCAG-5′ and 16s_926R 3′-CCGTCAATTCCTTTRAGTTT-5′. Only samples that contained amplifiable rDNA were submitted for sequencing. Illumina sequencing was performed by a commercial vendor (Novogene Europe, Cambridge, UK). Sequencing libraries were generated by amplification of the V3-V4 region and index-coded using the NEBNext Ultra DNA Library Pre®Kit for Illumina according to the manufacturer’s instructions. Library quality was assessed using a Qubit@ 2.0 fluorometer (Thermo Scientific) and an Agilent Bioanalyzer 2100 system. Finally, the library was sequenced on an Illumina platform, and 250 base pair (bp) paired-end reads were generated. One sample from a healthy participant could not be analyzed successfully.

### Bioinformatics analyses

The resulting sequences were trimmed, and paired-end reads were fused using FLASH (V1.2.7, [14]). After quality control, sequences were dereplicated, and chimeras were removed (UCHIME [15] using the GOLD database). Operational taxon units (OTUs) were clustered at 97% similarity using USEARCH. Taxonomic annotations were performed using the RDP classifier (version 2.2, http://sourceforge.net/projects/rdp-classifier/) with the GreenGenes database (http://greengenes.lbl.gov/cgi-bin/nph-index.cgi) as a reference. Downstream analyses were performed with R software (v 3.6.3) [16] and the packages vegan [17] and phyloseq [18]. Data were standardized to contain the same number of sequences per sample, using the sample with the lowest number as a reference.

Measures of alpha and beta diversity were used to analyze differences in the composition of gut microbiota between the study groups.

### Alpha diversity

was assessed by the Shannon and CHAO1 indices as well as the abundance-based coverage estimator (ACE), which were calculated with the functions diversity or estimateR (R package vegan).

### Beta diversity

In contrast to alpha diversity, which looks at the number of species observed in an environment, beta diversity describes the partitioning of biodiversity between environments, e.g., the number of species common to two environments. In the case of the present study, the similarity or difference in the composition of the gut microbiome between the groups studied was assessed [19]. The between-sample diversity (beta diversity) was analyzed using the Bray-Curtis distance. Beta diversity was calculated with vegdist using method “bray” which calculates the Bray-Curtis dissimilarity.

### Statistical analysis

Since studies on the difference in the gut microbiome in diabetic patients with depression are lacking, the study was designed with an explorative character. Accordingly, no power analysis could be performed to estimate the appropriate sample size.

Statistical analyses of sociodemographic, psychometric, and medical variables were performed with IBM SPSS Statistics for Windows, version 26 [20]. One-way analysis of variance (ANOVA) was used to compare continuous variables among the three participant groups, and multivariate analysis of variance (MANOVA) was used for the additional comparison of further subgroups. Bonferroni post hoc tests were performed to assess group differences in detail. Student’s *t* tests were used when the comparison involved only two groups. Differences in nominal scaled variables between groups were analyzed with *X*^*2*^ tests.

All analyses, including microbiota-related variables, were performed using R software (v 3.6.3). Variables indicating alpha diversity (ACE, CHAO1, Shannon index, number of species) were compared using the Kruskal-Wallis test. Correlations between psychometric data and alpha diversity were assessed with Pearson correlation coefficients. The Wilcoxon signed-rank test was performed to calculate differences in microbiota composition with groups divided by median splits. Only taxa with a median number > 10 in both groups were reported. Beta diversity was analyzed using the vegan package (adonis function) to compare Bray-Curtis distances in permutation analysis of variance (PERMOVA). Post hoc Tukey tests were performed to assess group differences in detail. For all analyses, a *p* value < 0.05 was considered significant.

### Quality assurance and ethics

A data validation plan was established describing the data management procedures, and data entry from case report forms was manually double checked. To assure the validity of the depression diagnoses, we established rater training for the DIMD-short version interview. The study was approved by the Ethics Committee of the Medical Faculty of the Ruhr-University Bochum (project number 18–6345) and is in accordance with the Helsinki Declaration. All participants gave written informed consent to participate in the study.

## Results

The main results regarding the composition of the gut microbiome in the studied groups yielded clear and statistically significant differences in terms of both the alpha and beta diversity between the two diabetes groups (with, group B, or without, group A, depression) and the healthy control group C.

### Alpha diversity

With respect to alpha diversity, all measures and indices examined showed similar findings (see Table 1): groups A and B showed significantly higher alpha diversity than the healthy control group C (*p* < 0.001). No statistically significant differences were observed between participants with type 1 diabetes with (group B) versus without depression (group A). When variables that differed between the groups (BMI, years of higher education, smoking and thyroid disease) were introduced into the model, the effect of the study groups remained significant.

### Beta diversity

**Fig. 1:**
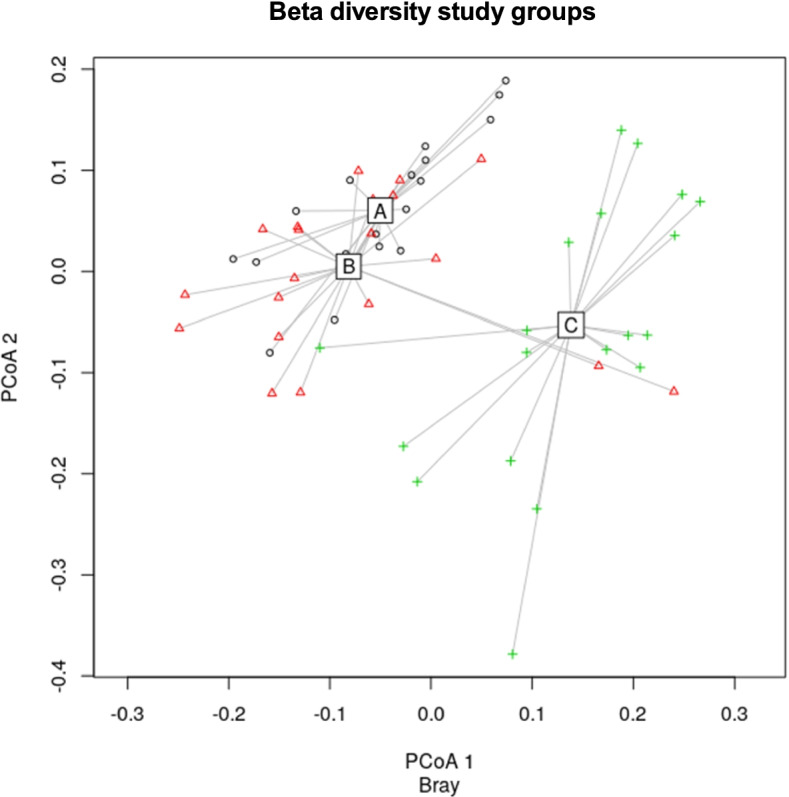
Significant differences in gut microbiota beta diversity (Bray Curtis distances) between study groups. Note. Participants with type 1 diabetes (A), participants with type 1 diabetes and comorbid depression (B) and healthy the control group (C) (the two principle coordinate axes (PCoA) are shown)

Principle coordinate analysis of beta diversity (Bray-Curtis dissimilarity) revealed a different composition of the microbiome of the control group C as compared to groups A and B (Fig. 1, *F* (2,56) = 11.165, *p* = 0.05).

Post hoc results (Tukey test) showed that both, participants with diabetes (Dif = 0.079, *p* < 0.001) and participants with diabetes and comorbid depression (Dif = 0.463, *p* < 0.05) were significantly different from the healthy group in terms of beta diversity. When variables that differed between the groups (BMI, years of higher education, smoking and thyroid disease) were introduced into the model, the effect of the study groups remained significant.

### Abundances of bacterial taxa (see Table 1 and Fig. 2)

Significant differences were observed at the phylum level for *Firmicutes, Bacteroidetes* and *Actinobacteria* between the studied groups. The abundance of *Firmicutes* was lower in the control group than in the group with diabetes and depression (*χ*^*2*^ (2) = 6.4105, z = 2.4616, *p* = 0.04). *Bacteroidetes* were more abundant in the control group than in the group with diabetes and depression (*χ*^*2*^ (2) = 5.9933, z = − 2.4406, *p* = 0.044) and *Actinobacteria* were more abundant in the control group than in the group with diabetes but without depression (*χ*^*2*^ (2) = 7.1343, z = − 2.6536, *p* = 0.024).

**Fig. 2:**
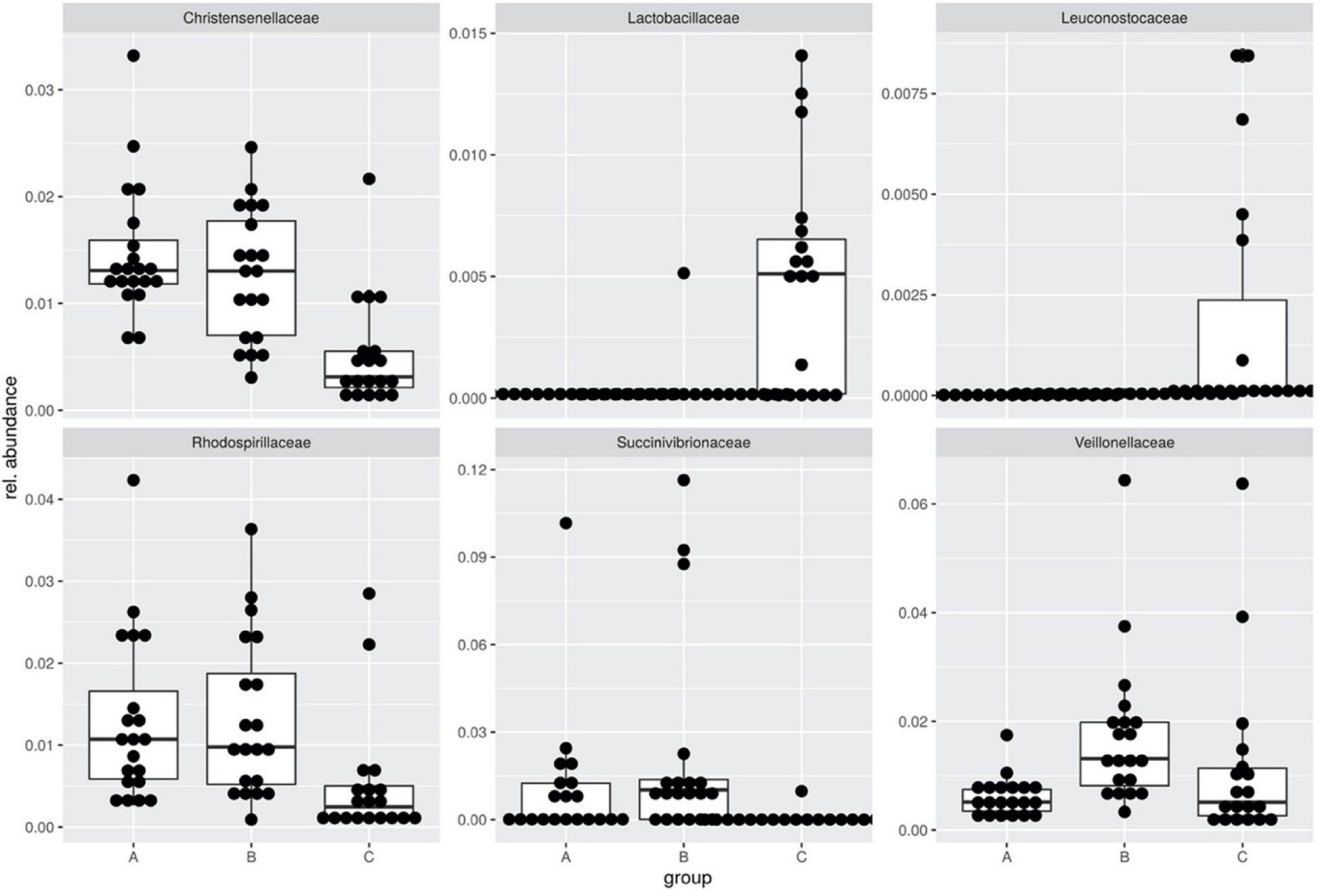
Significant differences in the abundance of bacterial families in the microbiota between study groups. Note. Participants with type 1 diabetes (A) and comorbid depression (B) and the healthy control group (C)

At the family level, the following differences were observed:

*Veillonellaceae (χ*^*2*^ (2) = 15.598, *p* < 0.05): Participants with type 1 diabetes and depression (group B) had a significantly higher abundance of *Veillonellaceae* than participants with type 1 diabetes without depression (group A; *z* = − 3.747, *p* < 0.001) and the healthy control group (*z* = 2.936, *p* ≤ 0.01).

*Lactobacillaceae* (*χ*^*2*^ (2) = 17.131, *p* < 0.0189): Participants in the control group had significantly higher abundances than participants in both diabetes groups (with depression: z = − 3.933, *p* < 0.001; without depression: z = − 3.124, *p* < 0.01).

*Leuconostocaceae* (*χ*^*2*^ (2) = 34.293, *p* < 3E-6): Again, participants in the control group had significantly higher abundances than participants in the diabetes groups (with depression: z = − 4.633, *p* < 0.001; without depression: z = − 5.451, *p* < 0.001).

Regarding *Proteobacteria*, statistically significant differences were observed with respect to *Succinivibrionaceae* (*χ*^*2*^ (2) = 31.444, *p* < 0.001) and *Rhodospirillaceae* (*χ*^*2*^ (2) = 14.781, *p* < 0.05). Post hoc tests demonstrated that for both bacterial taxa, participants with type 1 diabetes with (*Succinivibrionaceae*: *z* = − 4.633, *p* < 0.001; *Rhodospirillaceae*: *z* = 3.339, *p* < 0.001) or without (*Succinivibrionaceae*: *z* = − 5.451, *p* < 0.001; *Rhodospirillaceae*: *z* = 3.348, *p* < 0.01) depression had higher abundance than the healthy control group.

At genus level *Pediococcus* (family L*actobacillaceae*) and *Weissella* (also *Lactobacillaceae*) were more abundant in the control group (Kruskal-Wallis Χ^2^ = .40.9, df = 2, A:C z = − 6.02 *p* < 1e-4, B:C z = − 4.94 *p* < 1e-4 and Χ^2^ = 36,1, df = 2, A:C z = − 5.31 *p* < 1e-4, B:C z = − 5.13 *p* < 1e-4). *Coprococcus* (family *Lachnospiraceae*) was more abundant in groups A and B (Χ^2^ = 27.69, df = 2, A:C z = 5.12 *p* < 1e-4, B:C z = 3.67 *p* = 0.0004). In addition, the *Prevotellaceae*_NK3B31_group was less abundant in controls than in groups A and B (Χ^2^ = 28.97, df = 2, A:C z = 4.58, B:C z = 4.77, *p* < 1e-6). *Succinivibrio* was also more abundant in groups A and B compared to group C (Χ^2^ = 31.7, A:C z = 4.65 *p* < 1e-4, B:C z = 5.10 *p* < 1e-4). Also we found *Megasphaera* (family *Veillonellaceae*) to be more abundant in group B, i.e., patients with diabetes and depression (Χ^2^ = 15.22, A:B z = − 2.11 *p* = 0.052, B:C z = 3.89 *p* = 0.0002).

*Christensenellaceae* R7 group (*χ*^*2*^ (2) = 22.788, *p* < 0.001): A significantly higher abundance of this bacterial group was also observed in participants with type 1 diabetes both with and without depression than in control participants (z = 3.692, *p* < 0.001 and z = 4.491, *p* < 0.001, respectively).

### Sociodemographic variables of the study participants (see Table 1)

Analysis of sociodemographic differences between the study groups revealed a significantly lower number of participants with more than ten years of formal education in the group with type 1 diabetes and depression (*n* = 7/20, 35,0%) than in the type 1 diabetes without depression (*n* = 16/20, 80,0%; *p* < 0.05) and healthy control groups (*n* = 17/20, 85,0%; *p* < 0.01). In accordance with the parallelization of the groups, there were no statistically significant differences with respect to sex and age.

### Medical variables of the study participants

The body mass index was significantly higher in the type 1 diabetes with depression group than in the type 1 diabetes without depression (27.15 ± 4.18 vs. 23.76 ± 2.65; *p* < 0.01) and healthy control groups (27.15 ± 4.18 vs. 24.19 ± 2.72; *p* = 0.05). Participants with type 1 diabetes and comorbid depression were significantly more likely to report smoking than individuals in the healthy control group (*n* = 6/20, 30,0% vs. *n* = 1/20 5.0%; *p* < 0.05).

Regarding the variable “further somatic diseases”, which included various diseases, there were statistically significant differences between the group with type 1 diabetes and depression and the healthy control group (*n* = 18/20, 90,0% vs. *n* = 9/20, 40.0%; *p* < 0.01). Among the three most common diseases observed (arterial hypertension, thyroid disease, and hypercholesterolemia), only thyroid disease showed a statistically significant increase for the group with type 1 diabetes and depression with respect to the healthy control group (*n* = 11/20, 55% vs. *n* = 2/20, 10%; *p* < 0.01). No significant differences were found between the groups with respect to any other medical variables examined, including dietary habit.

### Psychological variables of the study participants

According to the inclusion criteria, there was a significantly stronger degree of depressive symptoms, as measured with the PHQ-9, in the group of patients with type 1 diabetes with depression than in the group of patients with type 1 diabetes without depression (14.15 ± 3.54 vs. 6.05 ± 3.75, *p* < 0.001) and the healthy control group (14.15 ± 3.54 vs. 1.80 ± 1.24, *p* < 0.001). The general stress level measured by the TICS-SSCS was also significantly more pronounced in the group with type 1 diabetes and depression than in both the group with type 1 diabetes without depression (27.80 ± 7.04 vs. 13.25 ± 9.78, *p* < 0.001) and the healthy control group (27.80 ± 7.04 vs. 8.15 ± 4.66, *p* < 0.001). Diabetes distress as measured with the PAID was severe in the group of participants with type 1 diabetes and depression and significantly higher than in the group of participants with type 1 diabetes without depression (41.81 ± 15.31 vs. 20.55 ± 17.28, *p* < 0.001).

## Discussion

Our study provided the first results on the extent to which comorbid depression is associated with specific changes in the gut microbiome in participants with type 1 diabetes and compared to healthy individuals. Participants with type 1 diabetes (groups A and B) showed higher alpha diversity than healthy individuals.

At least in newly diagnosed patients with type 1 diabetes, alpha diversity is often lower than in controls and the abundance of *Bacteroidetes* is higher [21, 22]. However, in two recent studies adult patients with type 1 diabetes and healthy controls had similar Shannon diversity [23].

In contrast, in rats put under chronic stress, lower alpha diversity has been found and *Bacteroidetes* were increased [24]. In patients with major depression, however, also increased alpha diversity and increased abundance of *Bacteroidetes* have been described [25]. Our results thus corroborate the conclusion of these authors that high alpha diversity may not always be beneficial. Also the notion that the abundance of a certain phylum is associated with disease may not always hold.

A detailed exploratory analysis of abundance at the family level revealed a higher abundance of *Veillonellaceae* in the group of patients with diabetes and depression. This substantiates existing findings [26] showing that *Veillonellaceae* are associated with diabetes. In addition, in rats under chronic stress, *Veillonellaceae* showed higher abundance than in controls [24]. We found one Genus with the family *Veillonellaceae*, *Megasphaera*, to show significantly higher abundance in patients with diabetes and depression. This genus has been found to be associated with Parkinson’s disease [27] and with large-artery atherosclerotic ischemic stroke and transient ischemic attack [28, 29] *Megasphaera* produces short chain fatty acids [30] which are often though of as beneficial; however, association of the genus with pathological conditions suggests that unknown factors that influence pathogenesis exist. Both groups with diabetes showed a higher abundance of *Christensenellaceae*, another *Firmicutes* bacterium, than the healthy control group. These bacteria have been found to be associated with affective disorders [31]. *Lactobacillaceae* and *Leuconostocaceae* (both order *Lactobacillales*) were virtually absent in the diabetic participants regardless of depression status and were present in the control group. Lower levels of this bacterial group have been previously described for patients with depression [32]. For *Rhodospirillaceae* and *Succinivibrionaceae* (both phylum *Proteobacteria*), the abundance was higher in participants with diabetes and lower in controls, supporting the view that this bacterial group is often associated with pathological situations (e.g., Alzheimer’s disease [33] and Behcet’s disease [34]). Intriguingly, there were low numbers of *Lactobacillales* and a higher abundance of some *Proteobacteria* in participants with diabetes. Generally, higher abundance of *Lactobacillales* (*Lactobacillaceae* and *Leuconostocaceae* are families of this order) is typically associated with healthier state [35, 36] whereas some *Proteobacteria* are ore often found to be associated with disease [37, 38] and even with mortality [39]. So these results would be in line with the literature.

A recent review on gut microbiota variations in patients with major depression revealed an increased relative abundance of the genus *Bifidobacterium* and a decreased relative abundance of *Faecalibacterium*. However, both the results of the included studies and their methodology revealed considerable heterogeneity [31]. Thus, our results are inconsistent with these findings, as we could not observe significant differences in the abundance of the genus *Bifidobacterium* in participants with depression (data not shown). One possible explanation for these findings could be that other studies investigating the microbiome in depressed patients included patients receiving antidepressant medication, which has been shown to affect the gut microbiome [32, 33].

### Limitations and strengths of the study

A fourth control group with depressed participants without diabetes would have contributed to the further methodological quality of the study. However, due to difficulties in recruiting depressed individuals who are not taking antidepressant medication, this control group was not included. A clear strength of our study is its robust methodology, including its control study design, an interview-based depression diagnosis, and a stringent control of confounding variables by the inclusion and exclusion criteria (e.g., antidepressant medication).

## Conclusions

In conclusion, our study can be viewed as supporting the hypothesis that the composition of the gut microbiome is associated with the development of depression in diabetes. To verify the robustness of the results, replication studies are now needed to understand the role of specific bacterial groups more thoroughly, particularly of the genus *Megasphaera*.

## Data Availability

The datasets generated during and/or analyzed during the current study are available from the corresponding author on reasonable request.

## Abbreviations

DIMD: Diagnostic Interview for Mental Disorders
DSM-5: Fifth Edition of the Diagnostic and Statistical Manual of Mental Disorders
EEG: Electroencephalography
LDEAP: Loudness-dependent auditory evoked potentials
OTUs: Operational taxon units
PAID: Problem Areas in Diabetes Questionnaire
PCR: Polymerase chain reaction
PHQ-9: Patient Health Questionnaire
StorAX: Stool transport tubes
TICS-SSCS: Trier Inventory of Chronic Stress
QIAamp: Fast DNA Stool Mini Kit
